# Recent advances in biosynthesis of bioactive compounds in traditional Chinese medicinal plants

**DOI:** 10.1007/s11434-015-0929-2

**Published:** 2015-11-02

**Authors:** Lei Yang, Changqing Yang, Chenyi Li, Qing Zhao, Ling Liu, Xin Fang, Xiao-Ya Chen

**Affiliations:** Plant Science Research Center, Shanghai Chenshan Botanical Garden, Shanghai Key Laboratory of Plant Functional Genomics and Resources, Shanghai, 201602 China; National Key Laboratory of Plant Molecular Genetics and National Center for Plant Gene Research, Institute of Plant Physiology and Ecology, Shanghai Institutes for Biological Sciences, Chinese Academy of Sciences, Shanghai, 200032 China; University of Chinese Academy of Sciences, Beijing, 100049 China

**Keywords:** Medicinal plant, Biosynthesis, Phenylpropanoid, Terpenoid, Alkaloid

## Abstract

Plants synthesize and accumulate large amount of specialized (or secondary) metabolites also known as natural products, which provide a rich source for modern pharmacy. In China, plants have been used in traditional medicine for thousands of years. Recent development of molecular biology, genomics and functional genomics as well as high-throughput analytical chemical technologies has greatly promoted the research on medicinal plants. In this article, we review recent advances in the elucidation of biosynthesis of specialized metabolites in medicinal plants, including phenylpropanoids, terpenoids and alkaloids. These natural products may share a common upstream pathway to form a limited numbers of common precursors, but are characteristic in distinct modifications leading to highly variable structures. Although this review is focused on traditional Chinese medicine, other plants with a great medicinal interest or potential are also discussed. Understanding of their biosynthesis processes is critical for producing these highly value molecules at large scale and low cost in microbes and will benefit to not only human health but also plant resource conservation.

## Introduction

China is rich in plant resources. Of the ~300,000 species of higher plants on the earth, around 10 % can be found in China. As in many other countries, people in China have used plants for treatment of diseases for thousands of years. *Compendium of Materia Medica* has been held in high esteem since it was first published in 1593, and this ancient encyclopedia of traditional Chinese medicine (TCM) described more than 1,000 species of plants. Plants produce a wealth of specialized (or secondary) metabolites also known as natural products, which are small molecular weight compounds with enormous structural diversity and show various biological activities. It is estimated that there are approximately 200,000 secondary metabolites in plant kingdom [1], which, based on biosynthetic origins, can be classified into three major categories: phenylpropanoids, terpenoids and alkaloids, plus a few other less abundant groups. The usage records of China’s ancient medical books, such as *Sheng Nong’s Herbal Classic*, *Huang Di’s Canon of Medicine* and *Compendium of Materia Medica*, already recognized that plant extracts contain active principles in treating illness and classified them into assumptive, intuitive or largely philosophic categories, such as cold, neutral or hot, toxic or nourishing. Over the past century, hunting the active ingredients has led to important findings, such as artemisinin for malaria, huperzine A for Alzheimer’s disease, ephedrine for cold and camptothecin for cancer, which were isolated from *Artemisia annua*, *Huperziaserrata*, *Ephedra sinica*, *Camptotheca acuminate*, respectively [2]. Very recently, tetrandrine, an alkaloid isolated from the TCM plant *Stephania tetrandra* previously used for reducing blood pressure, were reported to have the therapeutic efficacy against Ebola [3], and celastrol, a triterpene extracted from *Tripterygium Wilfordi*, has the potential as an anti-obesity agent [4]. These findings strongly support that TCMs are the reliable source for new therapies in treatment of lethally epidemic disease and long unsolved disease.

However, multi-classes of natural products are generated by each plant species. In addition, geographic distributions, growth conditions and harvesting seasons could significantly affect chemical compositions of the plant. Whereas one component may act as the active ingredient, the effects of a mixture of many ingredients are often uncertain and this has caused increasing concerns [5]; thus, the traditional practice of herbology has to face the challenges from modern medicine and the manufactures’ requirement.

While plant natural products continue to be a prime source for drug discovery and development, supply of these compounds is often curtailed due to limitation of natural resources and/or low contents in plant. The biotechnological platforms, such as metabolic engineering of effective plant and microbial production, are urgently needed to ensure that the supply of bioactive natural products is sustainable and environmentally friendly, rather than at the expense of resource exhaustion [6–9]. A prerequisite to these solutions is the understanding of the biosynthetic pathways of these specialized metabolites, in particular the cloning and identification of enzymes and the regulatory factors.

In the past two decades, the rapid development in genomics and high-throughput technologies of chemical analysis, in combination with molecular biology tools, has accelerated the research of medicinal plants. In this review, we summarize the recent advances in the elucidation of biosynthetic pathways of secondary metabolites in, not exclusively, TCM plants. Although alkaloids are probably the most important resource for drug discovery and biosynthesis of these amino acid-derived compounds has been investigated intensively, there are, surprisingly to some extent, relatively few studies of alkaloids from TCM plant; thus, this review is emphasized on phenylpropanoids and terpenoids. In addition to enzymes, transcription factors characterized from medicinal plants are also discussed.

## Phenylpropanoids

Phenylpropanoids, commonly found in plants, are derived from the six-carbon aromatic phenyl group and the three-carbon propene tail [10], and form a large group of specialized metabolites including monolignols, lignans, flavonoids, phenolic acids and stilbenes [11]. They serve as basic components of a number of structural polymers, as well as floral pigments, scent compounds or signaling molecules to mediate bio-interactions, phytoalexins against herbivores and pathogens, and protective components against ultraviolet light radiation and other abiotic stresses [12]. In many TCM plants, such as the plants of Lamiaceae, Fabaceae (Leguminasae) and Asteraceae, phenylpropanoids are also the bioactive principles (Table 1), which have been shown to act as anti‐oxidants, free radical scavengers, anti‐inflammatories and anticancer compounds [13].

**Table 1:** List of examples of TCM plants rich in phenylpropanoids

Plant species	Chinese name in Pin-yin	Family	Representative compounds
*Salvia miltiorrhiza*	Danshen	Lamiaceae	Salvianolic acid A, B and C
*Scutellaria baicalensis*	Huangqin	Lamiaceae	Baicalin, wogonin, scutellarin
*Glycyrrhiza uralensis*	Gancao	Leguminosae	Liquiritin, isoliquiritin, 7,4′-dihydroxyflavone
*Astragalus membranaceus*	Huangqi	Leguminosae	Calycosin-7-glucoside, ononin
*Sophora flavescens*	Kushen	Leguminosae	Sophoraflavecromane A, B, C
*Sophora tonkinensis*	Shandougen	Leguminosae	Sophoranone, sophoradin
*Pueraria lobata*	Ge	Leguminosae	Puerarin, daidzin, genistein
*Lonicera japonica*	Jinyinhua	Caprifoliaceae	Chlorogenic acid, luteolin
*Dendranthema morifolium*	Juhua	Asteraceae	Chlorogenic acid, acacetin-7-*O*-β-D-glucoside, apigenin-7-*O*-β-D-glucoside, and luteolin-7-*O*-β-D-glucoside
*Ginkgo biloba*	Yinxing	Ginkgoaceae	Ginkgetin, isoginkgetin
*Epimedium brevicornu*	Yinyanghuo	Berberidaceae	Icariine, icarisid
*Isatis indigotica*	Songlan	Brassicaceae	Lariciresinol

**Fig. 1:**
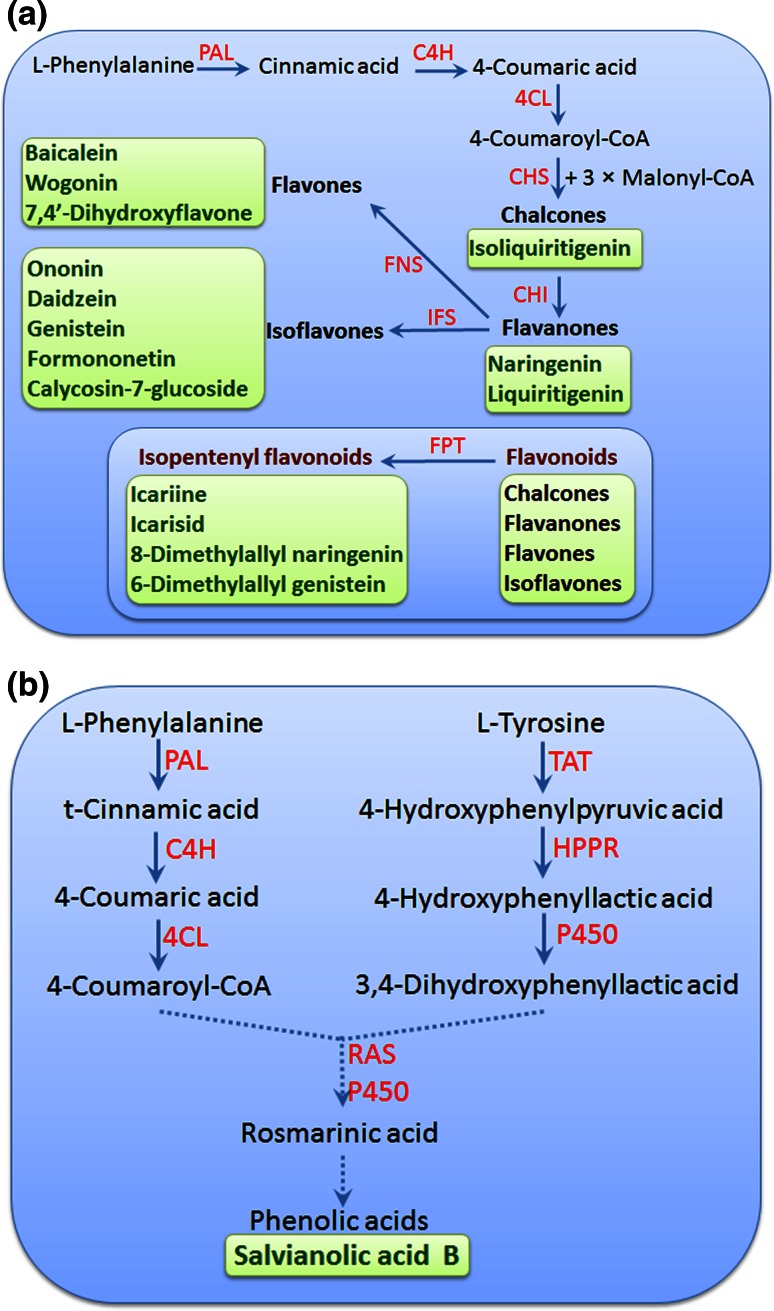
(Color online) Biosynthesis of phenylpropanoids in TCM plants. **a** Biosynthesis of flavonoids and isopentenyl flavonoids; **b** formation of phenolic acids from the l-phenylalanine- and the l-tyrosine-derived pathways in *Salvia miltiorrhiza*, a medicinal plant of Lamiaceae. Phenylpropanoids are mainly synthesized from phenylalanine via the “general phenylpropanoid pathway”, catalyzed by phenylalanine ammonialyase (PAL), cinnamate 4-hydroxylase (C4H) and p-coumaroyl coenzyme A ligase (4CL). The product of p-coumaroyl-CoA is used for the biosynthesis of flavonoids, isopentenyl flavonoids and phenolic acids. CHS, chalcone synthase; CHI, chalcone isomerase; FNS, flavone synthase; IFS, isoflavone synthase; FPT, flavonoid prenyltransferase; TAT, tyrosine aminotransferase; HPPR, 4-hydroxyphenylpyruvate reductase; RAS, rosmarinic acid synthase; P450, cytochrome P450 monooxygenase. Dotted lines represent multiple enzymatic catalyzed steps

The majority of phenylpropanoids are derived from phenylalanine. The first three steps are catalyzed by phenylalanine ammonia lyase (PAL), cinnamate 4-hydroxylase (C4H) and *p*-coumaroyl coenzyme A ligase (4CL), which are commonly referred as “general phenylpropanoid pathway” [14, 15]. The product of 4CL is used as precursor for the biosynthesis of various phenylpropanoids in plants (Fig. 1). Parts of phenylpropanoids are synthesized from l-tyrosine, and the transformation is more restricted, being mainly limited to members of several families. For instance, 3,4-dihydroxyphenyllactic acid, one precursor of rosmarinic acid, is synthesized from tyrosine-derived pathway in some species of Lamiaceae, such as *Salvia miltiorrhiza* [16, 17].

### Flavonoids

Flavonoids constitute a highly diverse class of secondary metabolites composed of more than 9,000 structures [18]. They are commonly found in land plants, including all vascular plants and some mosses [19]. Based on the aglycone core, they are generally further grouped into flavanones, flavones, flavonols, isoflavonoids, anthocyanins and proanthocyanidins. All flavonoids are basically derivatives of 1,3-diphenylpropan-1-one (C6–C3–C6), which is derived from the condensation of three malonyl-CoA molecules with one *p*-coumaroyl-CoA to form a chalcone intermediate [20]. Chalcone isomerase converts chalcone into flavanones, and respective enzymes transform flavanones to various flavones, isoflavones, dihydorflavonols, flavonols and anthoanidins. Every class of flavanones possesses the compounds with pharmaceutical activity and is widely used in folk medicines [21].

#### Flavanones and flavones

Two completely different flavone synthase (FNS) proteins have been found to catalyze the biosynthesis of flavones in plants. The first member of the FNS I type was identified from parsley (*Petroselinum crispum*) cell suspension cultures and classified as 2-oxoglutarate-dependent dioxygenase [22]. The FNS I cDNA was then cloned and functionally expressed in yeast [23], and it shares a high sequence identity to the flavanone 3-β-hydroxylase (FHT). Interestingly, characterized FNS I enzymes appear to be mainly in the family of Apiaceae [18, 24]. Molecular and phylogenetic analysis revealed a gene duplication of FHT, and a subsequent neofunctionalization occurred early in the development of the Apiaceae subfamilies [25]. Formation of most flavones in a wide range of plant species is catalyzed by FNS II, cytochrome P450 proteins of CYP93B subfamily. The FNS II activity was first demonstrated in extract of *Antirrhinum majus* flowers [26], and the cDNAs were then isolated from other plants, including *Perilla frutescens* (CYP93B6) [27] and *Gentiana triflora* [28].

*Glycyrrhiza uralensis* is one of the most popular TCM plants and also widely used in food flavoring. Although the sweeting agent of this plant is glycyrrhizin, a triterpenoid saponin [29], flavanones and flavones are also important components in its root, which include liquiritigenin, isoliquiritigenin and 7,4′-dihydroxyflavone [30]. A P450 enzyme from *Glycyrrhiza echinata*, CYP93B1, was identified as flavanone 2-hydroxylase (F2H), a member of FNS II [31]. The products, 2-hydroxyflavanones, were transformed into flavones in vitro in acid treatment, suggesting that an additional enzyme, probably a dehydratase, was involved in catalyzing the formation of flavones. A full-length cDNA of cytochrome P450 *CYP93C2* was isolated from the elicited *G. echinata* cells, which was shown to encode 2-hydroxyisoflavanone synthase [32].

The flavones baicalin and wogonoside, as well as their aglycones baicalein and wogonin, represent the dominant flavonoids in *Scutellaria baicalensis*, a perennial species of Lamiaceae and an important herb in Chinese traditional and clinical-orientated medicine. The flavones, such as baicalin and wogonin, are distinct for lacking a 4′-OH group but having a 6-OH group on their A-ring [33]. Genes encoding the upstream enzymes of the pathway, including PAL, C4H, 4CL, chalcone synthase (CHS) and chalcone isomerase (CHI), have been isolated [34, 35]. However, the enzymes committed to the formation of the *S. baicalensis*-type flavones remain unknown. It is also possible that specific enzyme isoforms are involved in the formation of cinnamoyl-CoA [36]. It has been reported that accumulation of these flavones was enhanced by jasmonate (JA) treatment, and a R2R3-MYB transcription factor, SbMYB8, was found involved in the regulation [37, 38].

#### Isoflavones

The isoflavones are well studied for their substantial health promoting benefits. They are found mainly in leguminous plants and are the major bioactive ingredients in soybean, *Astragalus*, *Pueraria lobata* [39]. Isoflavones are converted from flavanones by the isoflavone synthase (IFS). By using EST-based approach combined with enzymatic assays, P450s of CYP93C subfamily from soybean were shown to have such activities [40, 41]. Members of this subfamily with IFS activity were also reported in other leguminous plants, such as *Lotus japonicus* [42] and *Trifolium pratense* [43].

*Astragalus membranaceus*, a species of Fabaceae, has been used in TCM for thousands of years. *Astragaus* is considered an adaptogen because it is believed to help protect the body against stresses, including those of physical, mental or emotional [44, 45]. In China, *Astragalus* has been used to help patients with severe forms of heart disease in relieving symptoms, lowering cholesterol levels and improving heart function. Constituents of the *Astragalus* roots (radix astragali) include polysaccharides, triterpenoids (astragalosides) and isoflavones [46, 47]. Isoflavones such as calycosin-7-glucoside and ononin are considered the important active components in this medicine. Hairy root system of *Astragalus* was developed a long time ago to produce these ingredients [48, 49]. Research at molecular level in this plant is limited, but will help reveal the biosynthetic pathway in this leguminous medicinal plant [50].

*Pueraria lobata*, also a species of Fabaceae, is commonly known as “kudzu”. Puerariae radix, the dried root of the kudzu, has been used in China as herbal medicine for the prevention of cardiovascular disease and rehabilitation of stroke patients [51]. The major secondary metabolites accumulated in kudzu roots are isoflavones, including daidzein, genistein, formononetin and their glucosides Puerarin [52], among which the 8-*C*-glucoside of daidzein is considered the major active compound [53]. The co-occurrence of both *O*- and *C*-linked glycosides in root is of particular interests and worthy of further investigation. Using a functional genomics approach, He et al. identified enzymes associated with the isoflavone biosynthesis in kudzu roots, including 15 UDP-dependent glycosyltransferases (UGTs), among which one, GT04F14, exhibited the in vitro activity of glycosylation of a wide range of substrates, including coumarins, flavones, flavonols, and isoflavones. The isoflavones are converted region-specifically to their 7-*O*-glucosides, whereas *C*-glycosylation might take place at the 2,7,4′-trihydroxyisoflavanone precursor of daidzein, rather than directly on daidzein. Conceivably the intermediate 8-*C*-β-glucopyranosyl-2,7,4′-trihydroxyisoflavanone is converted to puerarin under in vivo conditions by the action of 2-hydroxyisoflavanone dehydratase (HID). A candidate gene encoding HID was identified from the EST library of kudzu root [54]. In addition, a partially purified preparation from kudzu root was shown to have the *C*-glucosyltransferase activity that converted isoliquiritigenin (2′,4′,4-trihydroxychalcone) and UDP-Glc to puerarin [55].

#### Isopentenyl flavonoids

Prenylation, the addition of prenyl groups, contributes to the diversification of flavonoids, and the occurrence of more than 1,000 prenylated flavonoids in plants has been recorded [56]. This prenylation represents the coupling process of the aromatic moiety from shikimate pathway and the prenyl (isoprenoid) chain from the isoprenoid pathways. Many prenylated flavonoids were identified as active components in medicinal plants and thus are of particular interests as lead compounds for drugs and functional food ingredients [57].

Species *Sophora*, family Fabaceae, are widely distributed in Asia. *Sophoraflavescens* has a long history of use in China, and the root, known as Ku Shen, is a typical TCM. It is used to dispel heat, dry dampness and eliminate intestinal parasites. It is thus administered in formulas for the treatment of dysentery and jaundice (damp-heat syndromes), edema and dysuria (dampness syndromes), and eczema and pruritis (damp-heat-wind syndromes). The *S. flavescens* prenyltransferase SfN8DT-1 is the first enzyme identified to be responsible for the prenylation of naringenin at the 8-position, with dimethylallyl diphosphate (DMAPP) as substrate [58]. Later, two new flavonoid prenyltransferases (FPTs) were isolated from *S. flavescens* at the molecular level: one is the isoflavone-specific prenyltransferase (SfG6DT) for the prenylation of the genistein at the 6-position and the other a chalcone-specific prenyltransferase designated as isoliquiritigenin dimethylallyltransferase (SfiLDT) [29].

Herba epimedii is prepared from the aerial parts of *Epimedium brevicornum* or *Epimedium sagittatum,* species of Berberidaceae. Herba epimedii contains various bioactive components and has been utilized extensively in China as the tonic and anti-rheumatic herb for thousands of years, and in the treatments of diseases such as impotence, frequency/urgency of urination, coronary heart disease, chronic bronchitis and neurasthenia [59, 60]. The isopentenyl flavonoids icariine and icarisid are the major active compounds [61]; however, their biosynthesis remains poorly understood [62]. Recently, Huang et al. isolated 12 structural genes and two putative transcription factors (TFs) in the flavonoid pathway. Transcriptional analysis revealed that two R2R3-MYB TFs (EsMYBA1 and EsMYBF1), together with a bHLH TF (EsGL3) and WD40 protein (EsTTG1), are probably involved in coordinated regulation of biosynthesis of the anthocyanins and the flavonol-derived bioactive components [63].

### Phenolic acids

*Salvia miltiorrhiza* is a perennial herb in the mint family (Lamiaceae). Its dried root or rhizome is called Danshen in TCM and was recorded in first pharmaceutical monograph *Shennong’s Classic of Materia Medica* (A.D. 102-200). *S. miltiorrhiza* has been cultivated throughout Eastern Asia and used to prevent and cure cardiovascular, cerebrovascular, hyperlipidemia and acute ischemic stroke diseases [64]. Both the hydrophilic and lipophilic components in *S. miltiorrhiza* are considered active ingredients. The hydrophilic compounds are mainly phenolic acids including rosmarinic acid, salvianolic acid B, lithospermic acid and dihydroxyphenyllactic acid or Danshensu, and they may also function as antioxidative, anti-bacterial and anti-viral reagents [65, 66].

The biosynthetic pathway for phenolic acids in *S. miltiorrhiza* is distinct and has attracted many interests. Labeling experiments using [ring-(13)C]-phenylalanine suggested two intermediates derived from the phenylalanine-derived general phenylpropanoid pathway and the tyrosine-derived pathway, respectively (Fig. 1): 4-coumaroyl-CoA and 3,4-dihydroxyphenyllactic acid (DHPL), which are coupled by a acyl-CoA-dependent acyltransferase BAHD family enzyme rosmarinic acid synthase (SmRAS) to form 4-coumaroyl-3′,4′-dihydroxyphenyllactic acid (4C-DHPL). The 3-hydroxyl group is introduced later in the pathway by a P450 monooxygenase (SmCYP98A14) to form rosmarinic acid (RA) [16]. This type of P450 was first reported in *Coleus blumei* (Lamiaceae), and it catalyzes the 3-hydroxylation of 4-coumaroyl-3′,4′-dihydroxyphenyllactate and the 3′-hydroxylation of caffeoyl-4′- hydroxyphenyllactate, in both cases forming rosmarinic acid [67]. Recent genome assembly to search the putative enzymes involved in biosynthesis of phenolics in *S. miltiorrhiza* revealed twenty-nine candidates, among which 15 were predicted in the phenylpropanoid pathway, seven in the tyrosine-derived pathway and six encoding putative hydroxycinnamoyltransferases [17].

## Terpenoids

Terpenoids are formed from sequential assembly of five-carbon building blocks (C_5_H_8_) called isoprene units. Accordingly, single or assemblies of two, three and four units constitute hemiterpenes, monoterpenes, sesquiterpenes and diterpenes, respectively. After the formation of the basic carbon skeletons, subsequent modifications, such as oxidation, reduction, isomerization and conjugation, lead to enormous numbers of structures, which represent the most abundant class of plant specialized metabolites, with more than 36,000 individual compounds [68].

**Fig. 2:**
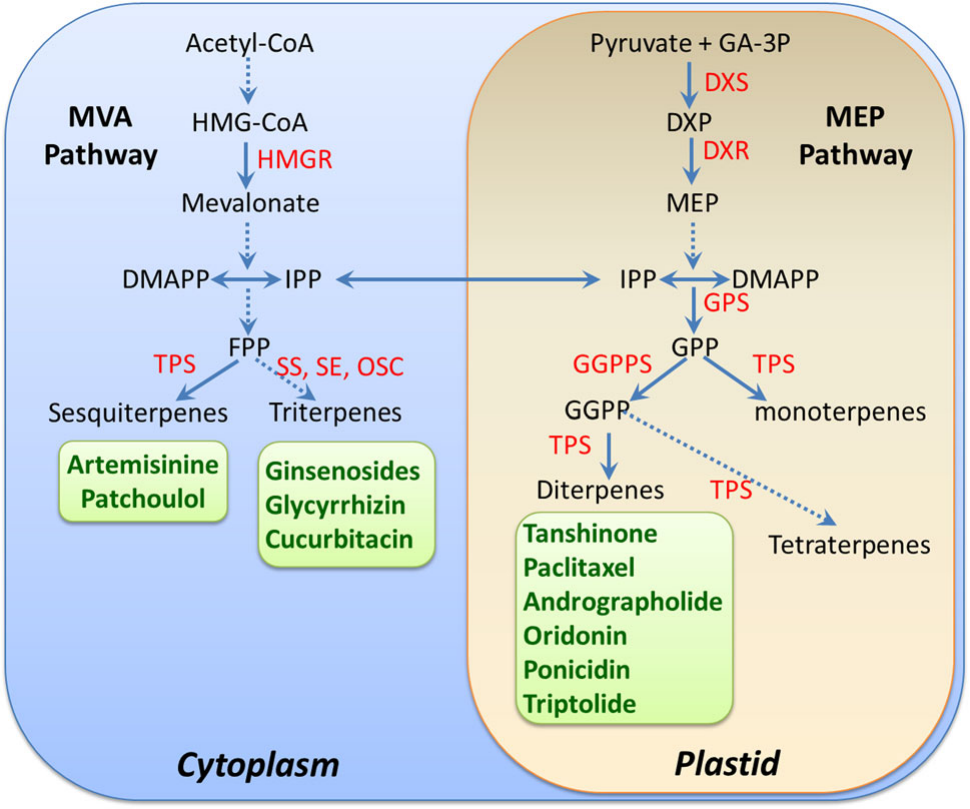
(Color online) Biosynthesis of terpenoids in TCM plants. Terpenoids are synthesized via the cytosol MVA pathway and plastid MEP pathway. Generally, isopentenyl diphosphate (IPP) and dimethylallyl diphosphate (DMAPP) synthesized from the MVA pathway are converted to farnesyl diphosphate (FPP) for the biosynthesis of sesquiterpenoids and triterpenoids, whereas those derived from the MEP pathway contribute to the formation of geranyl diphosphate (GPP) and geranylgeranyl diphosphate (GGPP) for biosynthesis of monoterpenoids, diterpenoids and tetraterpenoids. HMG-CoA, 3-hydroxy-3-methylglutaryl-CoA; MEP, 2-C-methyl-D-erythritol 4-phosphate; GGPP, geranylgeranyl diphosphate; HMGR, 3-hydroxy-3-methylglutaryl-CoA reductase; DXS, 1-deoxy-D-xylulose-5-phosphate synthase; DXR, 1-deoxy-D-xylulose-5-phosphate reductoisomerase; GPPS, geranyl diphosphate synthase; GPP, geranyl diphosphate; FPPS, farnesyl diphosphate synthase; GGPPS, geranylgeranyl diphosphate synthase; TPS, terpene synthase; SS, squalene synthase; SE, squalone epoxidase; OSC, oxidosqualene cyclase. Dotted lines represent multiple enzymatic catalyzed steps

In plant cells, the common precursors of terpenoids, isopentenyl diphosphate (IPP) and dimethylallyl diphosphate (DMAPP) are synthesized via two independent pathways: the cytosolic mevalonic acid (MVA) pathway that starts with the condensation of acetyl-CoA, and the plastid-localized methylerythritol phosphate (MEP) pathway that uses pyruvate and glyceraldehydes 3-phosphate as substrates (Fig. 2). The IPP and DMAPP are condensed into geranyl diphosphate (GPP, C_10_), farnesyl diphosphate (FPP, C_15_) and geranylgeranyl diphosphate (GGPP, C_20_) by the respective prenyltransferases and then converted to terpenes by terpene synthases (TPSs), which catalyze the critical step that determines the structures of terpen skeletons [69].

Generally, the cytosolic MVA pathway provides the precursor of FPP for the biosynthesis of sesquiterpenes and triterpenes, whereas the plastid MEP pathway is responsible for the biosynthesis of GPP and GGPP for mono-, di-, and tetra-terpenes [70]. Although cross-talk between these two spatially separated IPP pathways is prevalent, particularly in a direction from plastid to cytosol, our understanding of the molecular mechanism behind remains primitive.

### Sesquiterpenoids

Monoterpenoids (C_10_) and sesquiterpenoids (C_15_) are widely distributed in plants, and they are the common constituents of volatile compounds in flowers, fruits, stems and leaves, playing important roles in plant–environment interactions, many of them also possess great commercial value and some are used in pharmaceuticals.

#### Artemisinin

One of the most famous plant-sourced medicines is artemisinin, an endoperoxide sesquiterpene lactone isolated from *Artemisia annua* L., an annual herb of Asteraceae. Due to its effectiveness against drug-resistant cerebral malaria, it is the essential component of the combinational therapies recommended by the World Health Organization [8]. It has saved millions of lives globally, especially in developing countries. The 2011 Lasker DeBakey Clinical Research Award and the 2015 Nobel Prize in Physiology or Medicine honor the Chinese scientist Youyou Tu who made the important contribution to the discovery of artemisinin [71–73].

As a sesquiterpenoid, artemisinin is believed to be synthesized from the cytosolic MVA pathway. However, a recent report suggested that the MEP pathway may also contribute to its biosynthesis. GPP, which is synthesized in plastids, can be transported to cytoplasm, forming FPP with the addition of another IPP unit [74]. The FPP is converted to the artemisinin skeleton by amorpha-4,11-diene synthase (ADS), a sesquiterpene synthase [75], and then oxidated by the cytochrome P450 CYP71AV1. When expressed in *Saccharomyces cerevisiae*, CYP71AV1 catalyzed the continuous oxidation of amorpha-4,11-diene into artemisinic alcohol and artemisinic aldehyde [76], with significantly increased production of artemisinic acid and artemisinic aldehyde when co-expressed with a cytochrome b5 (CYB5) in yeast [8]. The artemisinic aldehyde ∆11(13) reductase (Dbr2), a double-bond reductase, catalyzes the formation of dihydroartemisinic aldehyde [77], which is further converted into dihydroartemisinic acid by aldehyde dehydrogenase 1 (ALDH1) [78]. Moreover, an additional alcohol dehydrogenase (ADH1) was also found to be involved in the oxidation of amorpha-4,11-diene to artemisinic acid, with specificity toward artemisinic alcohol in *A. annua* plants [8].

Several transcription factors have been shown to participate in the regulation of artemisinin biosynthesis [79]. Two jasmonate responsive AP2/ERF proteins, AaERF1 and AaERF2, were found to up-regulate the transcription of *ADS* and *CYP71AV1* genes, by binding to the CRTDREHVCBF2 (CBF2) and RAV1AAT (RAA) motifs present in their promoters [80]. A WRKY family transcription factor, AaWRKY1, was demonstrated to be capable of binding to the W-box in the *ADS* promoter and involved in the regulation of artemisinin biosynthesis [81].

A deep sequencing on the transcriptome of *A. annua* to identify genes and markers for fast-track breeding was performed, and a detailed genetic map with nine linkage groups was built. Replicated field trials resulted in a quantitative trait loci (QTL) map that accounts for a significant amount of the variation in key traits controlling artemisinin yield, and positive QTLs in parents of new high-yielding hybrids were enriched, which made it available to convert *A. annua* into a robust crop [82]. Ma et al. [83] recently reported an integrated approach combining metabolomics, transcriptomics and gene function analyses to characterize gene-to-terpene and terpene pathway scenarios in a self-pollinating variety of *A. annua*. Forty-seven genes that mapped to the terpenes biosynthesis pathway were identified by sequence mining, and such metabolites-transcriptome network associated with different tissues is fundamental to metabolic engineering to artemisinin.

#### Patchoulol

Patchouli (*Pogostemon cablin*), a perennial herbaceous species of Lamiaceae, is not only a fragrant plant producing patchouli oil for cosmetics industry, but also a medicinal plant for the treatment of medical ailments, such as removing dampness, relieving summer heat and exterior syndrome, and serving as an anti-emetic and appetite stimulant [84]. The patchouli oil is composed of sesquiterpenoids dominated by (-)-patchoulol. The sesquiterpene synthase, patchoulol synthase, was firstly purified from patchouli leaves by chromatofocusing, anion exchange, gel permeation and hydroxylapatite chromatography [85]. Then, its cDNA was cloned and the recombinant patchoulol synthase was shown to produce patchoulol as the major product, plus at least 13 additional sesquiterpenes [86].

Patchouli oil in leaves accumulates with plant age: The content is low at juvenile stage and increases during plant growth and reaches a high level in mature plant. The microRNA156 (miR156)-targeted SQUAMOSA promoter binding protein-like (SPL) factors, which function as the major plant age cue in regulating developmental phase transition and flowering, play a key role in the age-dependent progressive up-regulation of the *patchoulol synthase* gene expression, and the patchouli oil biosynthesis. Interestingly, expression of a miR156-resistant form of SPL not only accelerated plant maturation but also promoted patchouli oil production [87].

### Diterpenoids

Certain groups of diterpenoids (C_20_), such as gibberellins, are regulators (phytohormones) of plant growth and development. Many other specialized diterpenoids, like tanshinone from *Salvia miltiorrhiza* and taxol from *Taxus*, are highly valuable in medicine. A few more examples include: stevioside, extracted from *Stevia rebaudiana* of Asteraceae, is a natural sweetest [88–90]; adenanthin, from the leaves of *Rabdosia adenantha*, induces differentiation of acute promyelocytic leukemia (APL) cells [91]; oridonin, from Lamiaceae plants *Isodon rubescens* and *Isodon amethystoides*, is a potential compound for molecular target-based therapy of leukemia [92]; and triptolide, a highly oxygenated diterpene isolated from *Tripterygium wilfordii*, was shown to have anti-leukemic activity [93].

#### Tanshinone

Besides the phenolic acids discussed above, tanshinones are another class of active diterpenoid compounds of *S. miltiorrhiza*, which include tanshinone I, tanshinone IIA, cryptotanshinone and dihydrotanshinone I. They are all abietane-type derivatives, among which tanshinone IIA is considered to be an important bioactive component in protecting cardiovascular system [94, 95], and tanshinone I was reported to be an apoptosis inducer and display anticancer activities [95].

As diterpenoid compounds, tanshinones are expected to be traced to the plastid MEP pathway, and their biosynthesis starts from the conversion of geranylgeranyl diphosphate (GGPP) to ent-copalyl diphosphate (CPP) and then to miltiradiene. The subsequent extensively structural tailing converts miltiradiene to cryptotanshinone, tanshinone I, tanshinone IIA or tanshinone IIB [96].

Based on sequence homology, enzymes shared by other diterpenoid biosynthesis have been characterized [96, 97]. To date, two enzymes specifically committed to the tanshinone biosynthetic pathway have been identified: the kaurene synthase-like (SmKSL), a diterpene synthase that utilizes CPP as substrate to produce miltiradiene [96], and a P450 monooxygenase CYP76AH1 which transforms miltiradiene to ferruginol [98], both representing the mile-stone achievement in the research of TCM plant. Recently, functional divergence of SmCPSs and SmKSLs was reported, which specified the roles of individual CPSs in tanshinone production in different tissues, including SmCPS1 in roots and SmCPS2 in aerial part, and SmCPS4 and SmKSL2 were found to oxidize ent-13-epi-manoyl in floral sepals, and the conserved SmCPS5 involved in the plant growth hormone gibberellin biosynthesis. This study is a typical example of how the evolutionary diversification of diterpenoids in plants in molecular level [99].

With the rapid development of sequencing technologies, several transcriptome datasets and the draft genome of *S. miltiorrhiza* have been reported. For examples, the cDNA library of whole plant contained 10,228 ESTs [100], the transcriptome of nearly entire growing cycle generated by Illumina revealed 56,774 unigenes [101], and the searching of the draft genome resulted in 40 putative genes encoding enzymes involved in the biosynthesis of universal isoprene precursors of IPP and DMAPP [102]. Genes encoding cytochrome P450 monooxygenases, dehydrogenases and reductases, as well as several groups of transcription factors were predicted to be involved in tanshinone biosynthesis by comparative analysis of transcriptomes generated from different tissues [103]. Recently, next-generation sequencing (NGS) and single-molecule real-time (SMRT) sequencing were combined to generate a more complete/full-length set of *S. miltiorrhiza* transcriptome, which provides a valuable resource for further investigation of tanshinone biosynthesis [104].

Organ- or tissue-specific patterns are common feature observed in biosynthesis and accumulation of specialized metabolites, as well as the expression patterns of corresponding genes [105–107]. Tanshinones are actively synthesized and stored in roots, whereas only a low or trace amount was detected in aerial organs like leaves [108]. Moreover, both the accumulation and the expression of the related genes of tanshinones in hairy root cultures can be induced by biotic elicitors, such as the carbohydrate fraction of yeast extract, and phytohormones of salicylic acid and jasmonate [97, 109–114]. Further investigation can be directed to the characterization of the signaling components and transcription factors that regulate the diterpenoid biosynthesis in *S. miltiorrhiza*.

#### Taxol (paclitaxel)

Taxol (paclitaxel) is a diterpenoid isolated from the bark of *Taxus* trees. The anti-mitotic and cytotoxic properties of taxol are derived from its activity in disrupting normal tubulin dynamics, leading to dysfunction of microtubules [115]. Fourteen enzymes involved in taxol biosynthesis have been identified, they are geranylgeranyl diphosphate synthase [116], taxadiene synthase [117], taxadien-5α-ol-*O*-acetyl transferase [118], taxane 2α-*O*-benzoyltransferase [119], baccatin III: 3-animo-3phenylpropanoyltransferase [120], 10-deacetylbacctin III-10-*O*-acetyltransferase [121], 3′-*N*-debenzoyl-2′-deoxytaxol *N*-benzoyltransferase [122], taxane 5-alpha hydroxylase [123], taxane 10-alpha hydroxylase [124], taxane 13-alpha hydroxylase [125], taxane 2-alpha hydroxylase [126], taxane 7-alpha hydroxylase [127], taxane 14-alpha hydroxylase [128] and phenylalanine aminomutase [129].

In addition to elucidation of the biosynthetic enzymes, progresses have been made in identification of transcription factors involved in taxol biosynthesis, which include members of the AP2 and WRKY families [130]. A recent report showed that the bHLH transcription factors of TcJAMYC1, TcJAMYC2 and TcJAMYC4 act as negative regulators of taxol biosynthesis *in T. cuspidata* cultured cells [131].

Due to the extremely low content of taxol (at ppm level) in plant, it requires massive harvesting to obtain sufficient amounts of the drug; thus, productions by total synthesis, semi-synthesis, tissue or cell cultures, endophytic fungal fermentation and more recently metabolic engineering and synthetic biology have attracted great interests [132]. Precursors of taxol biosynthesis have been produced in *Escherichia coli* [7] and *Saccharomyces cerevisiae* [123, 133], and the integration of parts (modules) of the whole pathway in separate organisms cultured together led to the combination of production of taxadiene in *E. coli* and oxygenation of taxadiene by *S. cerevisiae* [9].

### Triterpenoids

Triterpenoids are cyclization product of squalene which is condensed by two molecules of FPP. In general, triterpenoids are formed from MVA pathway in cytoplasm, as sesquiterpenoids.

#### Ginsenosides

Ginseng, the root of *Panax ginseng*, is one of the oldest traditional medicines and is widely regarded as a tonic in East Asia [88–90]. The principle bioactive constituents of Ginseng are ginsenosides, a group of tetra- or pentacyclic triterpene glycosides belonging to saponins [134]. The clinical and pharmacological activities of ginsenosides include anti-diabetic, anticancer, anti-amestic hypoglycemic, radioprotective, immunomodulatory, neuroprotective and anti-stress [135–139]. More than 40 ginsenosides have been isolated from the white and the red ginseng, and they show different biological activities based on their structural differences [140]. Generally, the major pharmacologically active ginsenosides belong to tetracyclic dammarane- and pentacyclic oleanane-type triterpene saponins [141].

The common precursor of ginsenosides is squalene, which is formed by condensation of two FPPs with squalene synthase (SS) [135, 142, 143]. In Ginseng, squalene is converted into dammarenediol-II by squalone epoxidase (SE). The cyclization of 2,3-oxidosqualene can result in two different type of triterpenoids: dammarane and oleanane type. Ginsenosides belonging to dammarane-type triterpenoids are biosynthesized from 2,3-oxidosqualene by dammarenediol synthase (DS) to form dammarenediol-II [144], whereas the biosynthesis of oleanane-type ginsenosides is started by β-amyrin synthase (PNY1) that transforms 2,3-oxidosqualene into β-amyrin [145, 146]. SS is considered a rate-limiting enzyme in the pathway and catalyzes the initial biosynthetic step for both steroids and triterpenoids [147]. PgPDR, a member of ABC transporters, was found to be involved in the ginsenosides accumulation upon MeJA induction [148].

#### Cucurbitacins

Cucurbitacins, conferring a bitter taste in cucurbits such as cucumber, melon, watermelon, squash, and pumpkin, belong to a class of highly oxidized tetracyclic triterpenoids mainly found in the plant of Cucurbitaceae family, in which *Gynostemma pentaphyllum*, *Hemsleya chinesis*, *Siraitia grosvenorii* and *Bolbostemma paniculatum* are well-known TCM plants. Recent studies suggest that cucurbitacins repress cancer cell progression [149] and inhibit neuroblastoma cell proliferation through up-regulation of PETN (phosphatase and tensin homolog) [150]. By genome-wide association study based on the genome variation map of 115 diverse cucumber lines, the gene of Csa6G088690 (Bi) encoding oxidosqualene cyclase is found to be correlated to the cucurbitacin C (CuC) biosynthesis. Co-expression and co-regulation studies revealed a 9-gene module responsible for CucC biosynthesis, of which, four enzymes, including Bi, two P450s and one ACT, were characterized. Moreover, two bHLH transcription factors, Bl (bitter leaf) and Bt (bitter fruit), were found to directly regulate the expression of 9-gene module in cucumber leaf and fruit, respectively. During the cucumber domestication, mutations occurred within Bt promoter region which decreased its expression in the fruit tissue which may have been selected and fixed and resulted in nonbitter fruit we eat nowadays [151].

#### Glycyrrhizin

The roots and stolons of *Glycyrrhiza* plants (*G.uralensis* and *G. glabra*) contain a large amount of oleanane-type triterpenoid glycyrrhizin. It is not only used worldwide as a natural sweetener and flavoring additive due to its sweet taste, but also exhibit a wide range of pharmacological activities, including anti-inflammatory [152], immunomodulatory [153], anti-ulcer [154], anti-allergy [155], and anti-viral activity [156–158].

From *G. glabra*, genes that encode enzymes responsible for triterpene skeleton formation, including the squalene synthase (SS) and β-amyrin synthase (bAS), were isolated [159, 160]. Later biosynthesis steps of glycyrrhizin involve a series of oxidative reactions at positions C-11 and C-30 and glucuronylation of the C-3 hydroxyl group. Enzymes that catalyze the oxidation steps have been found to be cytochrome P450 monooxygenases. One of them, CYP88D6, was characterized to catalyze the sequential two-step oxidation of β-amyrin at C-11 to produce 11-oxo-β-amyrin by both in vitro assay with recombinant protein and co-expression with β-amyrin synthase in yeast [161]. Another P450, CYP72A154, was identified to be responsible for three sequential oxidations at C-30 to transform 11-oxo-β-amyrin to glycyrrhetinic acid, a glycyrrhizin aglycone [162]. Both CYP88D6 and CYP72A154 transcripts were detected in the roots and stolons, but not in the leaves or stems, which is consistent with the accumulation pattern of glycyrrhizin in planta [161, 162].

## Alkaloids

Alkaloids are a group of nitrogen-containing compounds with basic properties, most of which are derivatives of amino acids [163–166]. Biosynthesis of alkaloids usually starts from modification of amino acids, mostly decarboxylation or deamination, and undergoes further steps like methylation, hydroxylation and oxidation, and/or coupled with other compounds. There are over 12,000 alkaloids that have been identified from plants. Although widely distributed in plants, they are particularly enriched in certain families, such as Solanaceae, Manispermaceae, Papaveraceae, Berberidaceae and Fabaceae (Table 2).

**Table 2:** List of examples of TCM plants rich in terpenoids

Plant species	Chinese name in Pin-yin	Family	Representative compounds
*Pogostemon cablin*	Guanghuoxiang	Lamiaceae	Patchoulol
*Artemisia annua*	Huanghuahao or Qinghao	Asteraceae	Artemisinine
*Salvia miltiorrhiza*	Danshen	Lamiaceae	Tanshinone
*Taxus chinensis*	Hongdoushan	Taxaceae	Paclitaxel
*Andrographis paniculata*	Chuanxinlian	Acanthaceae	Andrographolide
*Isodon rubescens*	Donglingcao	Lamiaceae	Oridonin, ponicidin
*Isodon amethystoides*	Xiangchacai	Lamiaceae	Oridonin, ponicidin
*Tripterygium wilfordii*	Leigongteng	Celastraceae	Triptolide
*Panax ginseng*	Ginsen or Renshen	Araliaceae	Ginsenosides
*Panax notoginseng*	Sanqi	Araliaceae	Notoginsenosides
*Radix liquiritiae*	Gancao	Fabaceae	Glycyrrhizin
*Dioscorea polystachya*	Shuyu	Dioscoreaceae	Dioscin

It is noteworthy that the most of alkaloids display bioactivities to certain degrees, often derived from their nitrogen-containing properties. Not surprisingly, alkaloids constitute the major portion of drugs both in history and nowadays. The discovery and isolation of morphine from the opium poppy (*Papaver somniferum*) by Friedrich Sertürner in 1806 is a milestone in the history of pharmacy. Investigations of biosynthesis of natural alkaloids such as morphinan, vindoline and noscapine have been intensive and led to the complete elucidation of the pathway [167–170], and increasing alkaloid biosynthesis in plant through co-expression of enzymes genes was also reported [114]. Unfortunately, although alkaloids with TCM background like camptothecin, higenamine, huperzine A and tetrandrine have been used in pharmacy, reports of their biosynthesis are relatively rare. We list in Table 3 several typical alkaloids in TCM plants, and the relevant references. Various aspects on the alkaloid biosynthesis, regulation and metabolites trafficking can be found in review articles [178–183]. Without doubt more efforts are needed to study alkaloids in TCM plants to further explore their biological activities and facilitate their usage.

**Table 3:** List of examples of TCM plants rich in alkaloids

Plant species	Chinese name in Pin-yin	Family	Representative compounds	References
*Camptotheca acuminate*	Xishu	Cornaceae	Camptothecin	[171–173]
*Coptis chinensis*	Huanglian	Ranunculaceae	Berberine	[174, 175]
*Isatis indigotica*	Songlan	Brassicaceae	Isatin, indigotin	[176]
*Baphicacanthus cusia*	Baalan	Acanthaceae	Isatin, indigotin	[177]

## Perspective

Unlike model plant or staple crops, medicinal plants often lack a well-studied genetic background and a high-quality genome sequence. Due to the recently developed high-throughput sequencing technologies, it is possible to generate transcriptomic data of medicinal plants in a short time at an affordable cost. Comparative analysis of chemical constituents, transcriptomes and correlation of spatial and temporal patterns of gene expressions with those of metabolite accumulation have led to the identification of candidate genes of the biosynthesis pathway [184]. GWS combined with metabolomics analysis (mGWAS) provides a powerful platform which screens a large number of accessions simultaneously to understand genetic contributions to the metabolic diversity [185, 186].

Throughout the history, herbal plants are an integral part of our lives. In addition to curing illness, they are grown in elegant gardens, provide natural fragrance, delicate accessories and stimulate appetite. The biosynthesis of metabolites in medicinal plants is complex and specialized and involves many sequence-similar but functionally diverged enzymes. With the fast development of new technologies of analytical chemistry, bioinformatics and synthetic biology, more and more achievements will be made in this genomic or post-genomic era and bring us better life.
